# Biodegradable composites with antibiotics and growth factors for dual release kinetics

**DOI:** 10.1007/s10856-024-06809-8

**Published:** 2024-07-29

**Authors:** Michael Seidenstuecker, Julian Hess, Anna Baghnavi, Hagen Schmal, Diana Voigt, Hermann O. Mayr

**Affiliations:** 1 G.E.R.N. Center of Tissue Replacement, Regeneration & Neogenesis, Department of Orthopedics and Trauma Surgery, Medical Center—Albert-Ludwigs-University of Freiburg, Faculty of Medicine, Albert-Ludwigs-University of Freiburg, Engesser Str. 4, 79108 Freiburg, Germany; 2 Department of Orthopedics and Trauma Surgery, Medical Center—Albert-Ludwigs-University of Freiburg, Faculty of Medicine, Albert-Ludwigs-University of Freiburg, Hugstetter Straße 55, 79106 Freiburg, Germany; 3 FILK Freiberg Institute gGmbH, Meissner Ring 1-5, 09599 Freiberg, Germany

## Abstract

Bone infections are still a major problem in surgery. To avoid severe side effects of systemically administered antibiotics, local antibiotic therapy is increasingly being considered. Using a pressure-based method developed in our group, microporous β-TCP ceramics, which had previously been characterized, were loaded with 2% w/v alginate containing 50 mg/mL clindamycin and 10 µg/mL rhBMP-2. Release experiments were then carried out over 28 days with changes of liquid at defined times (1, 2, 3, 6, 9, 14, 21 and 28d). The released concentrations of clindamycin were determined by HPLC and those of rhBMP-2 by ELISA. Continuous release (anomalous transport) of clindamycin and uniform release (Fick’s diffusion) of BMP-2 were determined. The composites were biocompatible (live/dead, WST-I and LDH) and the released concentrations were all antimicrobially active against *Staph. aureus*. The results were very promising and clindamycin was detected in concentrations above the MIC as well as a constant rhBMP-2 release over the entire study period. Biocompatibility was also not impaired by either the antibiotic or the BMP-2. This promising approach can therefore be seen as an alternative to the common treatment with PMMA chains containing gentamycin, as the new composite is completely biodegradable and no second operation is necessary for removal or replacement.

**Graphical Abstract:**

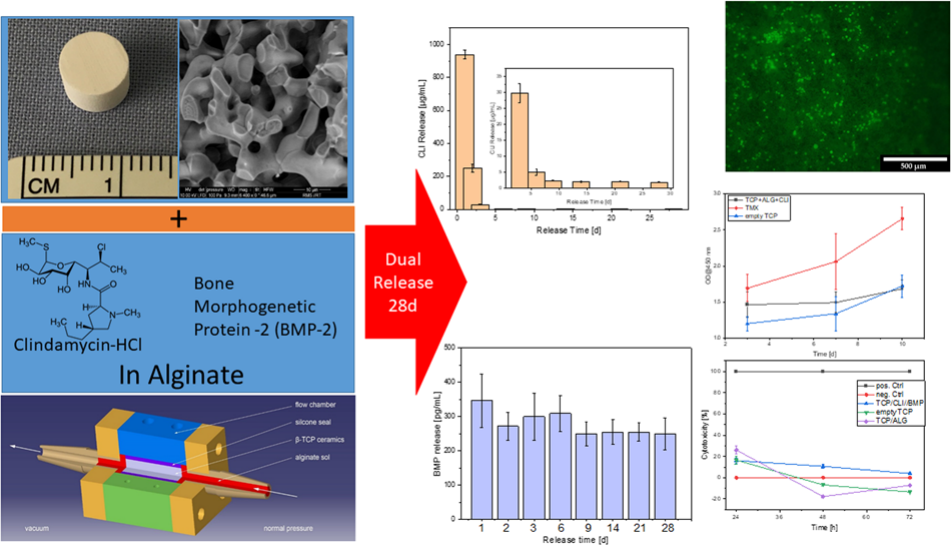

## Introduction

Periprosthetic joint infection (PJI) refers to a post-operative infection in the tissue around an implanted artificial joint in the human body. This infection can occur in any artificial joint, i.e., in hip, knee, elbow or ankle joints. Total hip and knee arthroplasties (THA, TKA) are successful and frequently performed operations with a prosthesis survival rate of up to 93% (THA) and 77% (TKA) after 15 years and 82% (THA) and 70% (TKA) after 25 years [1]. According to Eurostat and the Agency for Healthcare Research and Quality, THAs and TKAs are among the ten most commonly performed operations in both Europe and the USA. The numbers vary in European countries, with Germany and Switzerland performing more than 300 THAs per 100,000 population, while in the USA, hospitals perform 219 TKAs and 184 THAs per 100,000 population [2–4]. Despite improved safety standards, reports from the American College of Surgeons’ National Surgery Quality Improvement Program show that 4.2% of THAs and 5.55% of TKAs have complications within 30 days of surgery, with PJI being the most common [5, 6]. Infection rates vary by joint location and can lead to higher revision rates, which not only increases healthcare costs but also jeopardizes patient well-being [7, 8]. *Staphylococcus aureus* (*Staph. aureus*) is considered the main cause of PJI, therefore elimination or inhibition of initial growth is crucial in implant-associated infections [9, 10]. Treatment of acute PJI requires rapid surgical debridement and microbiologic testing, followed by 4–6 weeks of systemic or local antibiotic therapy [11, 12]. A locally applied approach involves the use of carrier systems such as non-degradable antibiotic-filled polymethyl methacrylate (PMMA) in bone cements or degradable solutions such as collagen sponges, lactic acid polymers, calcium phosphate polymers and autogenous bone grafts [13–18]. Such approaches allow high local antibiotic concentrations while systemic side effects can be avoided. Due to the fact that the population is getting older and older (every second person in Europe is older than 45 [19]) as well as changing requirement profiles such as sports into old age, it can be assumed that the demand for hip (up to 40%) and knee (up to 45%) endoprostheses will increase over the next three decades. The prevention and effective treatment of PJI is of crucial importance [20, 21].

PMMA beads, in particular, have some disadvantages, including incomplete release [22, 23], a limited range of miscible antibiotics and the risks associated with performing two operations. Calcium phosphate ceramics are well established as an implant material and their degradability is influenced by water solubility, among other factors, with the ratio of calcium to phosphate being of critical importance. Hydroxyapatite has a Ca/P ratio of 1.67, while other calcium phosphate ceramics such as β-tricalcium phosphate (β-TCP) with a more favorable ratio of 1.5 enable faster degradability [24]. In addition to water solubility, porosity also plays a significant role in the in vivo resorption of the ceramic material [25, 26]. These pores provide access for bone cells to migrate. The degree of porosity is controversial discussed, one group prefers macro pores [27] the other micro pores [28]. Porous ceramics allow the release of active substances [29, 30] at the defect site. There are various methods for loading porous ceramics with additives such as antibiotics and drugs. The way in which these active substances are introduced is of particular importance for their release. One method is to spray the active substances onto the surface. Another method is to incubate the ceramic in an aqueous solution containing antibiotics for a certain period of time [31–34]. The subsequent drying phase can be carried out in an oven at a maximum of 50 °C in order not to destroy the antibiotics [32, 33, 35]. Both air and vacuum drying are described in the literature [36–38]. As this is an adhesive load, these methods are generally used for short-term drug release [39]. In order to prolong the release, the shaped bodies loaded with active ingredients can also be encapsulated, similar to what is done in the pharmaceutical industry in the production of tablets and capsules. After the loading process, a layer is applied as a diffusion barrier, which can be done by dipping or spraying [40]. An alternative method is sol–gel preparation [33, 38, 41], in which tricalcium phosphate [42–44] or hydroxyapatite [45] in powder form [41, 46] or as sintered scaffold [47–49] is added to the polymer solutions together with the antibiotics. In a previous work [48], we were able to show that long-term release of a drug (vancomycin) over 28d is possible using alginate in β-TCP. In the present work, sintered ceramic cylinders made of β-TCP with a total porosity of 40% and a pore size of 5 µm are to be loaded with alginate. The alginate should serve as a diffusion barrier and contain both clindamycin (CLI) and bone morphogenetic protein 2 (BMP-2). This is an approach that has only been taken by us so far. The novelty of this idea is the simultaneous dual release of antibiotic and growth factor with two targets: (1) release of CLI to fight an infection, e.g., of the bone, and (2) release of BMP-2 to promote bone healing. We want to show that this is possible and that there are no interactions between the two drugs, although both are present in the alginate gel.

## Materials and methods

### Manufacturing process of β-TCP ceramics

The ceramic materials were manufactured following the previously outlined procedure [48, 50]. A mixture of 80 g α-tricalcium phosphate and 20 g tricalcium phosphate (Art. No. 102143, Merck; a blend of apatite and some calcium hydrogen phosphate) was combined with 60.0 ± 0.2 g of a solution containing 0.2 M Na_2_HPO_4_ and 1% polyacrylic acid (Art. No. 81132, Fluka; Mw = 5.1 kDa). The mixture was stirred, and the resulting paste was poured into plastic syringes with the tips removed (*Ø* = 23 mm). After 45 min, the paste solidified, and it was subsequently covered with 10 mL of PBS 7.4 solution (Art. No. P5368, Sigma) and incubated at 60 °C for 3 days. The samples, having a diameter of 23 mm and a length of 70 mm, were dried and sintered at 1250 °C for 4 h, with a heating and cooling rate of 1 °C/min. The cylinders were then trimmed to a length of 25 mm and a diameter of 10 mm. Following this, the samples were washed in ethanol to eliminate residual particles and calcined at 900 °C to eliminate all organic residues. Prior to use, the ceramics were shortened to cylinders with a length of 6 mm and washed once more. Finally, the samples underwent sterilization at 200 °C for 4 h.

### Characterization of the β-TCP

The structure was analyzed using ESEM (FEI Quanta 250 FEG, Hillsboro, USA) and the pore size distribution was determined using µCT (Scanco Micro-CT 50, Bruettisellen, Switzerland) and mercury porosimetry (Porotec Pascal 140/440, Hofheim, Germany). The parameters of the µCT were 90 kV, 4 W, 44 µA with a resolution of 2 µm and an integration time of 5000 s. The pore sizes were determined using Pascal 140 porosimeters in the size range 1000–1.4 µm with a pressure increase of 0.1 kPa and with the Pascal 440 porosimeters for the size range 1.4 µm–1.8 nm with a pressure increase of 400 MPa. The composition of the β-TCP was determined using EDX (Oxford Instruments, Abingdon, UK) and XRD (Bruker D8 Advance, Billerica, USA). The EDX used an accelerating voltage of 12 kV and a livetime corrected measurement duration of 100 s. The measurement conditions of the XRD were Bragg-Bretantano geometry, Cu anode and secondary graphite monochromator, scintillation counter, 40 kv/40 mA, 1°2 theta/min, step size 0.02°2 theta.

### Hydrogel preparation

To prepare the hydrogels plasma sterilized alginate (Art. No. A2033, Sigma Aldrich (now MERCK), Darmstadt, Germany) 2.0% w/v was added to aqua bidest in solution along with BMP-2 (Art. No. 10426-HNAE, Sinobilogical, Beijing, China) and clindamycin hydrochloride (Art. No. PHR1159, Sigma Aldrich (now Merck), Darmstadt, Germany) and homogenized for at least 12 h. In the run-up to BMP-2 release, a model substance FITC-conjugated protein A (Art. No. 101011, ThermoFisher, Waltham, USA) was used, which had a similar size to BMP-2 and was easy to detect using FITC.

### Hydrogel characterization

#### GPC analysis

Gel permeation chromatography (GPC) analysis was conducted to ascertain the molar mass distribution and mean molar masses of the employed alginate A2033 (Sigma Aldrich, now Merck, Darmstadt, Germany). A 20 mg sample was dissolved in 10 mL of the eluent over a period of 2 days at room temperature. Prior to measurement, the solutions underwent filtration through a PTFE filter membrane with a porosity of 1 µm. Pullulan standards were utilized in the separation region of the column combination for calibration. The molar mass distributions and averages of the samples (both sterile and non-sterile) were computed using the strip method with the aid of the Pullulan calibration curve. The GPC analysis conditions are outlined in Table 1.

**Table 1:** Analysis conditions for GPC

Eluent	0.02 M phosphate buffer, pH 6.6 + 0.5 M NaCl aq.
Columns	PSS Suprema, 10 µm precolumn, ID 8.0 mm ×50 mm
	PSS Suprema, 10 µm 100, ID 8.0 mm × 300 mm
	PSS Suprema, 10 µm 3000, ID 8.0 mm × 300 mm
	PSS Suprema, 10 µm 3000, ID 8.0 mm × 300 mm
Column temperature	35 °C
Pump	PSS SECurity 1260 HPLC pump
Flow rate	1.0 mL/min
Injection system	PSS SECurity 1260 autosampler
Injection volume	50 µL
Sample concentration	2 mg/mL
Detector	PSS SECurity 1260 RI-Detector
Analysis	PSS WinGPC UniChrom Ver. 8.33

#### Rheology

The Malvern Kinexus lab+ KNX2110 rheometer (Malvern, UK) was used for the rheological investigations. The cone plate used (CP1/40 SR3033 SS) had a diameter of 40 mm and an angle of 1°. The distance to the fixed plate (PLS40 S2345 SS) was 23 µm. The measurements with frequency ramp were performed with a shear strain of 1% and a temperature of 25 °C in the range of 0.02–16 Hz.

### Loading the ceramics

**Fig. 1:**
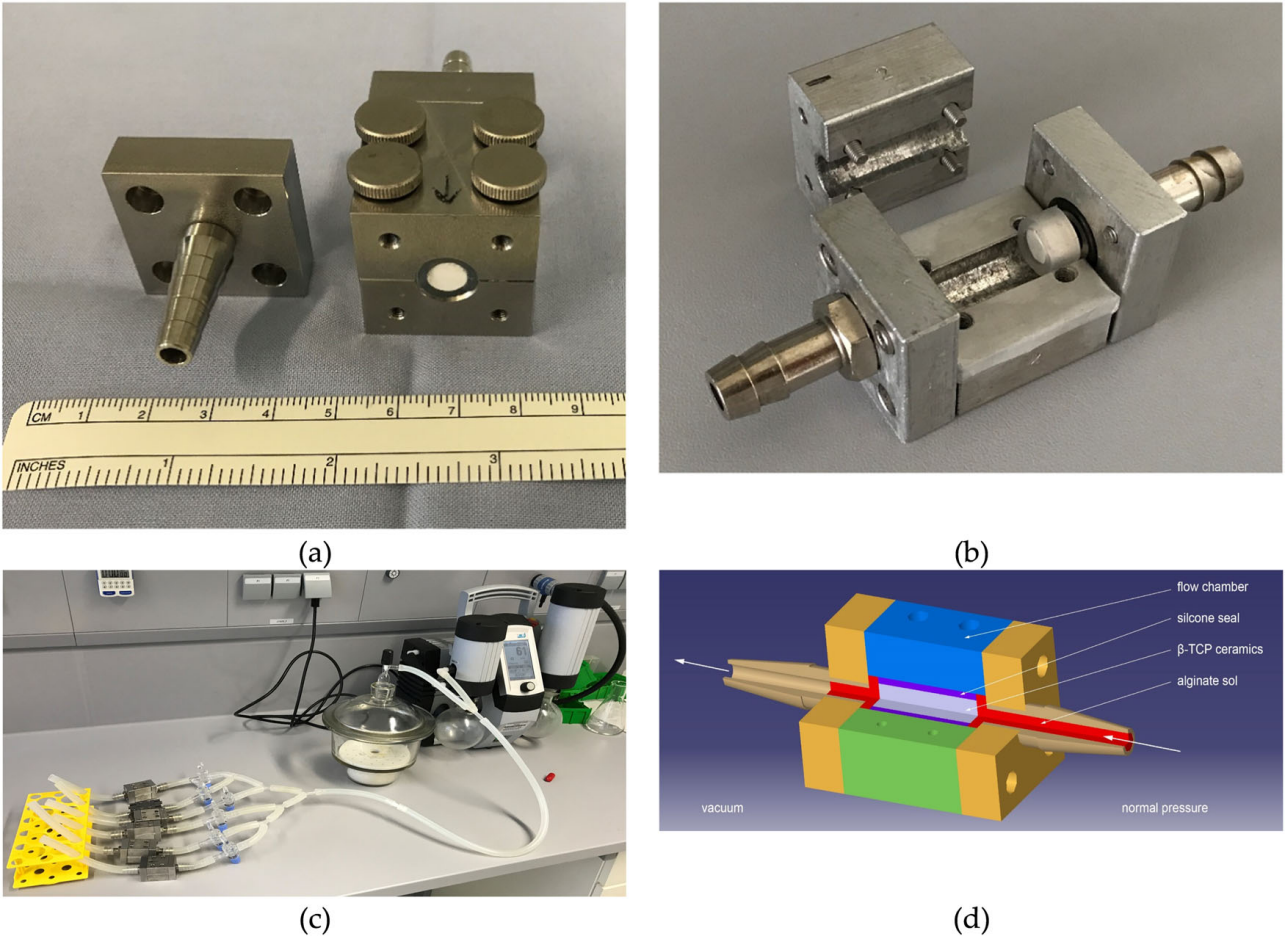
Overview of **a**, **b** flow chamber (arrow marks the flow direction), **c** load build-up and **d** load principle

The loading of β-TCP ceramics followed a procedure akin to that employed by Seidenstuecker et al. [47]. The ceramic scaffolds, which were cut and cleaned, were secured within a patented loading chamber (X5CrN18-10) and sealed using a silicone tube (stainless steel, inner diameter: 6 mm, outer diameter: 10 mm). Parafilm® M served as a seal between the two halves of the chamber. The assembled loading chamber, fastened with screws, featured connections on both ends: one side for later filling with hydrogel, and the other side connected to a fork, linking the system to a desiccator and a vacuum pump (Fig. 1). The desiccator maintained a vacuum of 50 mbar generated by the vacuum pump (KNF Neuberger SC920, Freiburg, Germany) and served as a water trap. Upon activating the pump, the two-way stopcock downstream of the loading chamber was opened to establish a vacuum within the ceramic. After 10 min, the alginate 2.0% w/v (A2033, Sigma Aldrich, sterile) in bidest water was introduced, and the front two-way stopcock was subsequently opened.

After loading, the ceramics were incubated in 30 mM CaCl_2_ (Art. No.: C1016, Sigma Aldrich (now Merck), Darmstadt, Germany) overnight solution for crosslinking. To prevent premature release from the composites, the CaCl_2_ solution contained the same concentration of CLI and BMP-2.

### Evaluating the loading of the ceramics

Instead of BMP-2, FITC-coupled protein A (Art. No. 101011, ThermoFisher, Waltham, USA) was employed as a surrogate for BMP-2 and served as a representative model substance. The use of FITC-coupled protein A offered the advantage of enabling detection through fluorescence microscopy, facilitating the assessment of loading. Subsequently, following the loading process, the ceramics were longitudinally broken open using a standard pair of pliers, and the cross-section was examined with a fluorescence microscope (Olympus BX51, Shinjuku, Japan). FITC fluorescence was visualized with excitation at 485 nm and emission at 514 nm. The loaded ceramic exhibited a pronounced green fluorescence, whereas an inadequately loaded area appeared faintly green or completely dark. The individual images were stitched together using Microsoft Image Composite Editor (version 2.0.3.0) due to the rapid decrease in fluorescence under illumination, necessitating swift image acquisition. In addition, the surface of the filled ceramics was analyzed using a 3D laser scanning microscope VK-X200 (Keyence, Osaka, Japan).

### Drug release experiments

All steps were to be carried out protected from light, as CLI was light-sensitive. Fifty milligrams per milliliter CLI were mixed into bidest water and stirred for at least 10 min. BMP-2 was added as 600 µL BMP-2 stock solution (corresponding to 300 µg BMP-2) to 29.4 mL CLI solution (50 mg/mL). To obtain a homogenous solution, the mixture was stirred for 1 h at 100 rpm. Finally, 2.0% w/v alginate A2033 sterile was added to the mixture of CLI and BMP-2 and stirred overnight. The loading of the ceramics was done as described in section 2.5.

#### Clindamycin release by HPLC

Before measurement, all samples underwent sterile filtration with a 0.2 µm pore size. For CLI analysis, HPLC (Shimadzu CBM-20A, CTO-20AC, DGU-20A5R, LC-20ADXR, Reservoir Tray, RF-20A, SIL-30AC, SPD-M20A IVDD, Kyoto, Japan; Macherey-Nagel precolumn EC 4/3 Nucleodur 300-5 C4ec, column EC 250/3 Nucleodur 300-5 C4ec, Duren, Germany) was conducted at a temperature of 25 °C, with a running time of 10 min and a flow rate of 0.66 mL/min. The mobile phase consisted of ACN and 25.08 mM Na_2_HPO_4_ (pH 3.5, adjusted with phosphoric acid) in a ratio of 29:71. The area under the curve was measured at a wavelength of 193 nm with a retention time of 3.49 ± 0.06 min.

#### BMP-2 release by ELISA

The Human BMP-2 ELISA Kit from Sino Biological (Art. No. KIT10426, Beijing, China) was utilized for ELISA analysis following the manufacturer’s instructions. The kit included a 96-well plate coated with capture antibody. After three washes with 300 µL wash buffer, 100 µL of each release sample was pipetted into the wells. Additionally, a BMP-2 standard (0–2500 pg/mL) was prepared and treated similarly to the samples. The pipetting of samples and the standard was completed within 15 min, followed by a 2-h incubation at room temperature. Subsequently, the wells were washed three times, and 100 µL of the detection antibody was added, incubating for 1 h at room temperature. After another three washes, 200 µL of the substrate solution was pipetted into the wells. Following a 20-min incubation at room temperature in darkness, the color reaction was halted with 50 µL of stop solution, and the absorbance was measured at 450 nm.

The release kinetics of all release fractions were determined by using the model of Ritger and Peppas [51]. The following formula was used:

$$\frac{{{{M}}}_{{{t}}}}{{{{M}}}_{\infty }}={{{kt}}}^{{{n}}}$$

Using *M*_*t*_ to represent the mass of the released drug at time *t*, *M*_∞_ for the mass of the released drug as time approaches infinity, *k* as a constant that encompasses the characteristics of the macromolecular network system and the drug, and *n* as a diffusional coefficient indicating the transport mechanism [51].

### Biocompatibility

MG-63 cells (ATCC, CRL 1427) were used for all biocompatibility tests. All tests were performed with 25,000 cells/100 µL per composite. Ten identical composites were used for each biocompatibility test and all tests were repeated at least three times. Composites as well as empty ceramics and Thermanox Coverslips were examined. The Themanox served as a 2D control and the ceramics as a 3D control.

#### Live dead assay

On each composite, 100 µL of medium containing 25,000 cells/100 µL of MG-63 were pipetted. The well plates were incubated for 2 h at 37 °C with 5% CO_2_ saturation in an incubator. Following the initial 2 h, 1 mL of Dulbecco’s Modified Eagle Medium nutrient mixture (DMEM/F12) was added to each well. The well plates were then further incubated in the incubator for 3, 7 and 10 days. For the staining solution, 2 mL of DPBS (Art. No. 14190-094, Gibco, Grand Island, NE, USA) was combined with 4 µL of ethidium homodimer III solution (along with the calcein component of the Live/Dead Cell Staining Kit II, PromoCell, Heidelberg, Germany) in a Falcon tube (Greiner Bio-One International GmbH, Kremsmünster, Austria), following the manufacturer’s protocol (PromoCell). Subsequently, 1 µL of calcein dye was added after a thorough mixing of the staining solution. All procedures were conducted in the dark to prevent photobleaching of the staining solution and samples. To eliminate serum esterase activity, the medium was removed, and the cells were washed at specific time points. Staining was carried out according to a previously established protocol [34]. Evaluation was performed using an Olympus fluorescence microscope (BX51, Olympus, Osaka, Japan) at five different positions on the samples, at 5× and 10× magnification.

#### Cell proliferation assay (WST-I)

Water-soluble tetrazolium salt (WST-1) was used to determine the metabolic activity of the cells, which is strongly correlated with cell viability. After washing cells in the well plate with PBS twice, the cells were treated for 2 h with the WST-1 solution (triphenyl tetrazolium chloride) (WST-1 KIT, Roche Molecular Biochemicals, Switzerland). After this incubation period, formazan solution was formed, which was quantified spectrophotometrically with an ELISA plate reader.

#### Cytotoxicity assay (LDH)

In the lactate dehydrogenase (LDH) assay, cytotoxicity was determined by the quantification of LDH in the medium. A total of 100 µL of supernatant was added to 100 µL of reagent from the test kit (Cytotoxicity Detection Kit, Roche Molecular Biochemicals, Switzerland) onto a 96-well plate. The activity was determined by colorimetric measurement of the reduction in sodium pyruvate in the presence of NADH. TritonX (Art. No. X100-100 ML, Sigma Aldrich (now Merck), Darmstadt, Germany) was used as positive (dead cells) and cells without treatment as negative control.

### Determining the minimal inhibitory concentration

To demonstrate the ongoing microbial efficacy of CLI after 28 days, its minimum inhibitory concentration was assessed in accordance with ISO Standard 20776-1 and EUCAST [52]. Samples collected on release days 1, 2, 3, 6, 9, 14, 21 and 28 of CLI-BMP-release underwent testing. HPLC-determined antibiotic concentrations facilitated the examination of a dilution series with fixed concentrations for each release day, in addition to the original concentration. The initial concentration, prepared with distilled water, underwent subsequent dilution using Müller–Hinton–Bouillon (MHB) (BBL Mueller Hinton II Broth, Cation-Adjusted, Art. No. 298268, BD Biosciences, Franklin Lakes, USA). Alongside the samples, growth controls were established using 100 µL MHB plus 5 µL bacterial suspension, and blank values with pure MHB. Moreover, samples of ceramics loaded with ADA-gelatine without active ingredients were assessed to determine whether the low proportion of MHB in the initial preparation adversely affected bacterial growth and whether the ADA-gelatine gel alone exhibited antimicrobial properties. For comparison, these samples were subjected to a 1:1 dilution with MHB. Duplicate preparations were made for each sample. In the experiments, the standard strain of *Staph. aureus* ATCC 29213 was pre-incubated on CBA plates (Colombia Blood Agar; Oxoid™, Thermo Scientific, Waltham, USA) for 24 h and adjusted to McFarland 0.5 with physiological NaCl solution [53]. This corresponded to a bacterial count of 1 × 10^8^/milliliter. The bacterial suspension was diluted 1:10 with MHB to achieve a concentration of 1 × 10^7^ mL^−1^. This resulted in the required bacterial count of 5 × 10^5^ mL^−1^ when 5 µL of this bacterial suspension was mixed with 100 µL of the respective sample for the MIC test. For the inoculum control, 10 µL from a growth control was diluted 1:1000 in MHB, and 100 µL was plated out on Colombia blood agar beforehand. Thus, 50 CFU had to grow on the plate for the required bacterial count of 5 × 10^5^ mL^−1^. After incubation at 35 °C for 18 ± 2 h, bacterial growth was monitored, and the MIC was determined.

### Statistics

For statistical analysis, mean values and standard deviations were determined via sampling time point and an analysis of variance was collected using (ANOVA). The comparison of averages was done according to Fisher LSD. The statistical significance level was set at *p* < 0.05. Calculations were performed using OriginPro 2023 (OriginLabs, Northampton, MA, USA).

## Results

### Characterization of the β-TCP

**Fig. 2:**
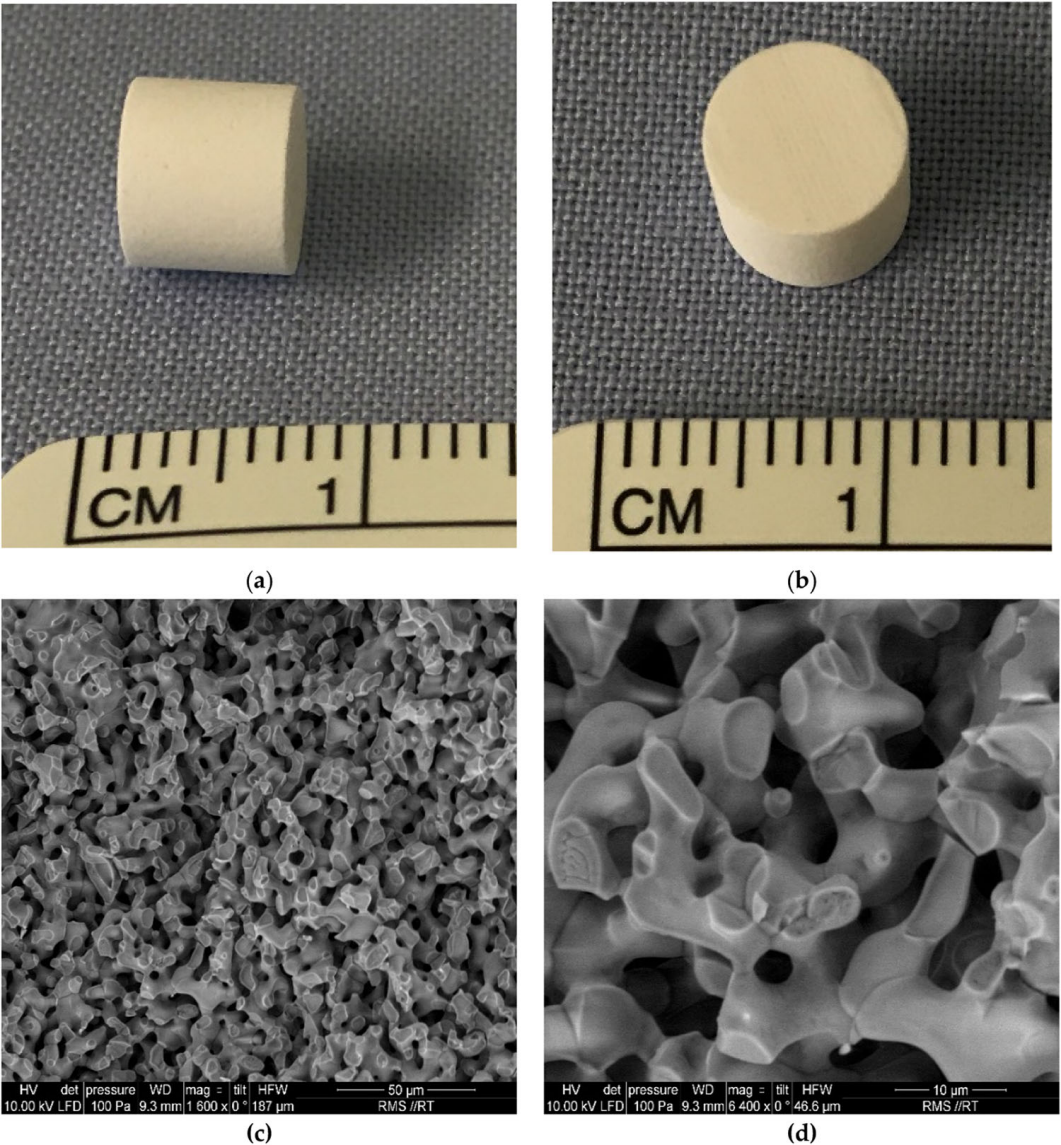
Overview of the β-TCP ceramics **a**, **b**: macroscopic; **c**, **d**: microscopic via ESEM

The β-TCP ceramics (Fig. 2a, b) exhibited a high level of porosity, with pore sizes ranging from 1 to 10 µm. Consistent with prior studies, the porous β-TCP ceramics were subjected to examination through ESEM, revealing a uniform pore structure as depicted in Fig. 2c, d.

**Fig. 3:**
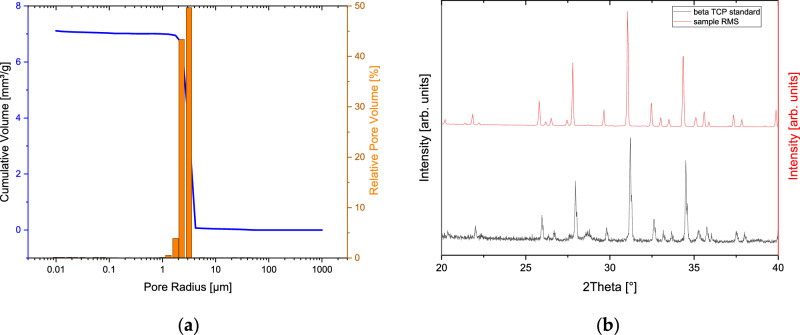
Pore size distribution (**a**) and XRD pattern (**b**) of the β-TCP ceramics. The XRD pattern was compared to the standard from the Joint Committee on Powder Diffraction (JCPDS) Database

The distribution of pore sizes was determined using the Pascal 140 and 440 mercury porosimeters, and the results were combined in a diagram presented in Fig. 3 (depicting the purple curve for Pascal 140 and the red curve for Pascal 440). The average pore radius measured 2.1 ± 0.3 µm. The overall porosity of the ceramic material was calculated to be 46.5 ± 3.6%. EDX analysis of the pure β-TCP samples revealed the presence of peaks corresponding only to O, Ca and P. The chemical composition of the sample exhibited peaks exclusively for oxygen, calcium and phosphorus. Quantification of the EDX spectrum indicated a composition of 60 at% Ca and 40 at% P, resulting in a calcium–phosphate ratio of 1.5. Calcium and phosphorus concentrations were determined (without conducting measurements with a pure substance to calibrate their parameters) based on theoretical k-factors. In Fig. 3b, the X-ray diffraction spectrum was compared with the standard for β-TCP from the Joint Committee on Powder Diffraction (JCPDS) Database, revealing a highly consistent match between the two spectra. Both the intensity and band positions of the reflections in the spectrum aligned closely with the standard, showing no unaccounted peaks that might indicate phase impurities, as well as no band broadening or alterations in individual reflexes. The increased noise observed in the sample compared to the standard was attributed to the coarse grinding of the sample. A subsequent Rietveld refinement analysis of the ceramics using Profex 3.0 (www.profex-xrd.org, freeware) revealed 99.6% β-TCP as well as traces of CPP resulting from the manufacturing process.

### Hydrogel characterization

#### GPC analysis

**Fig. 4:**
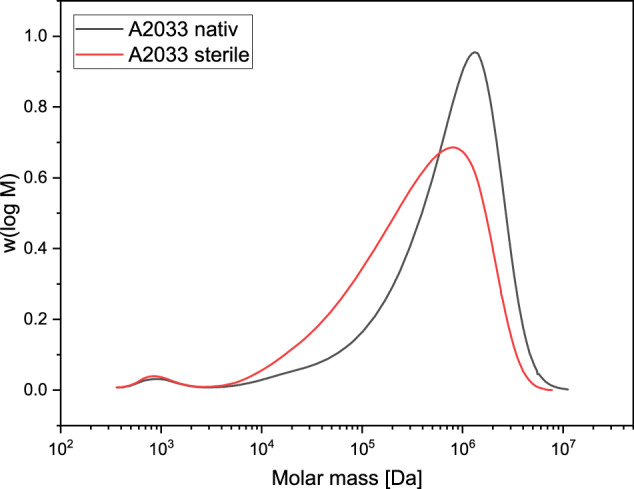
Molar mass distribution of native and sterile alginate A2033

The molar mass [Da] of the sterilized alginate decreased compared to the native alginate: Mn from 41,200 Da to 31,700 Da or Mw 1100 kDa to 668 kDa. The results are summarized in Table 2 and Fig. 4.

**Table 2:** GPC analysis of molar mass distribution for native and sterile alginate

Sample	Mn [Da]	Mw [Da]	Mz [Da]	PDI (=Mw/Mn)
A2033 native	41,200	1,100,000	2,090,000	26.76
A2033 sterile	31,700	668,000	1,510,000	21.08

#### Rheological analysis

**Fig. 5:**
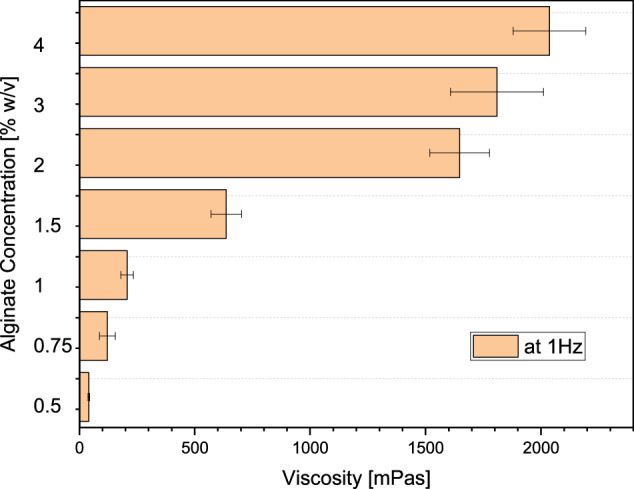
Dependence of viscosity on the concentration of sterile alginate; measured with Malvern KNX2110 rheometer (Malvern, UK) at 1 Hz

The measurements showed a viscosity of 1647 mPa·s for the sterile alginate A2033 with 2.0% w/v measured at 1 Hz. At this concentration, the ceramics could still be loaded within 10 min. This was no longer possible with the higher concentrations of sterile alginate and native alginate. This is also clearly shown by the overview of viscosities in Fig. 5.

### Evaluating the loading of the ceramics

**Fig. 6:**
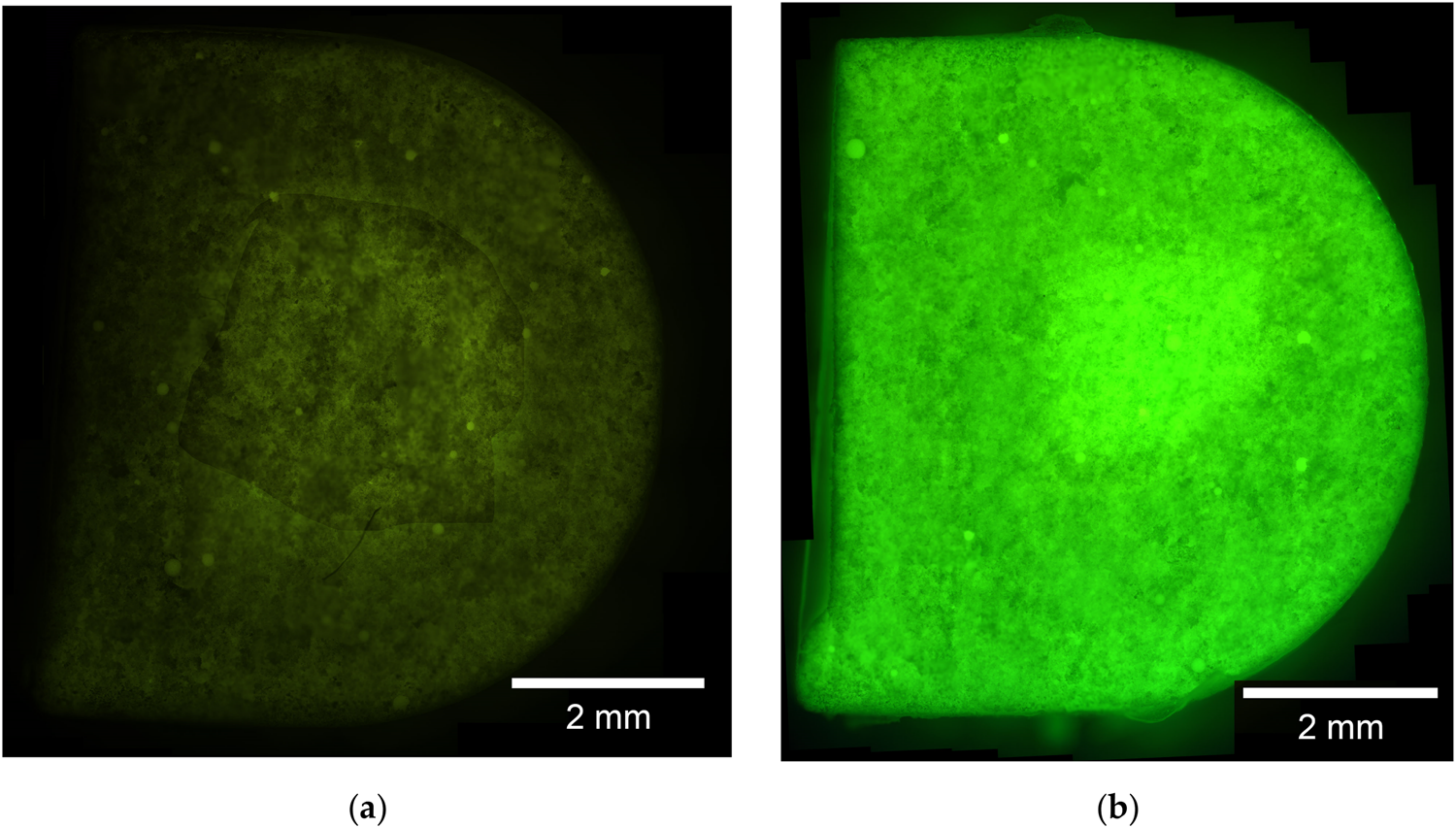
Fluorescence microscopy to measure the loading success; **a**: intrinsic fluorescence of the unloaded ceramic (500 ms exposure time), **b**: alginate and FITC-labeled protein A-filled ceramic (50 ms exposure time); images taken with Olympus BX51 and composited with MS ICE 3.0

The rounded ends of the ceramic dowel were used to determine the loading success; loading was carried out from the side with the straight end. The individual images from the fluorescence microscopy were combined using MS Image Composition Editor 3.0. Both the intrinsic fluorescence of the ceramic (in a dark khaki) and the fluorescence of the loaded ceramics with FITC-labeled protein A were recorded. Both are shown in Fig. 6. An exposure time of 50 ms was used to record the FITC prot A-filled ceramic. An exposure time of 500 ms was required to record the intrinsic fluorescence of the ceramic, which was not visible at 50 ms.

**Fig. 7:**
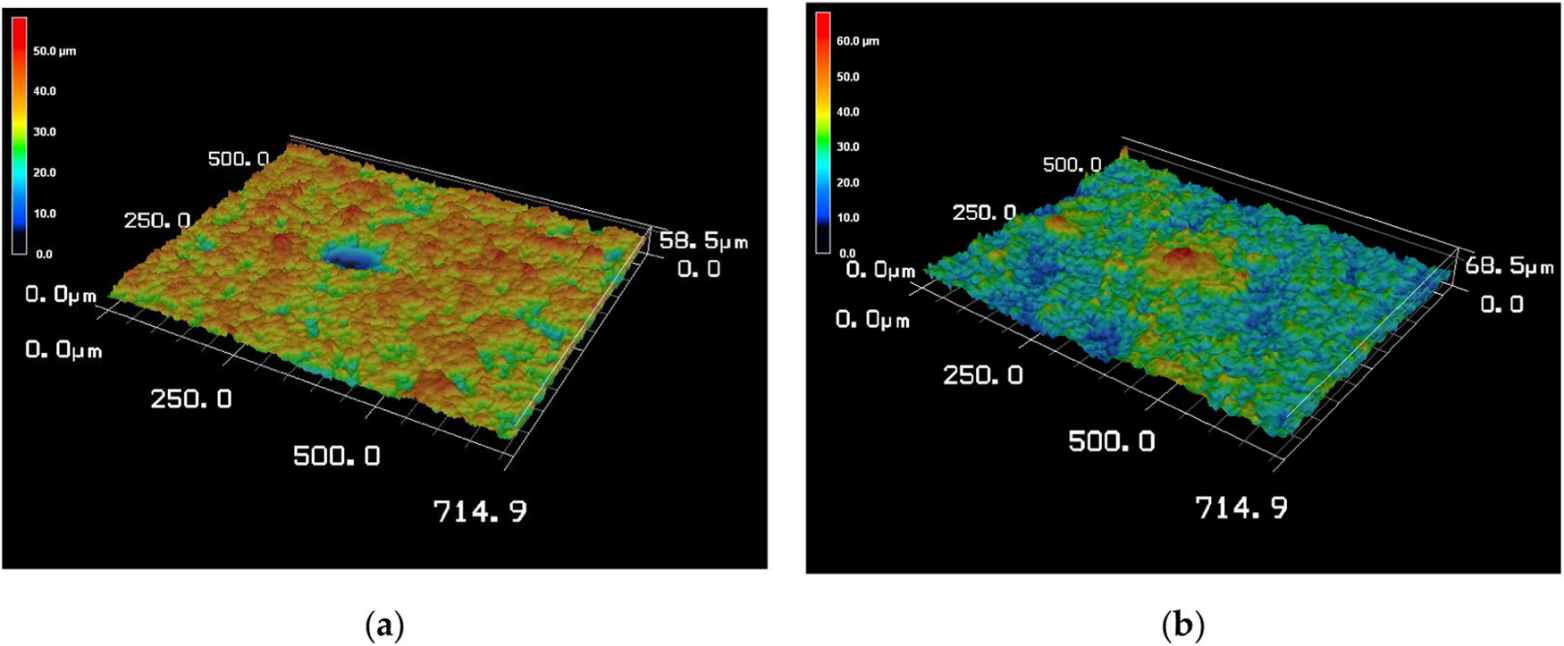
3DLSM images of **a**: empty ceramic with a clearly visible pore at the edge and **b**: an alginate-filled ceramic, images taken with Keyence VK-X200 3D laser scanning microscope with 400× magnification

After the core had been filled using fluorescence microscopy, the filling at the edge was to be checked using 3D laser scanning microscopy (3DLSM). The surfaces of the filled ceramics were analyzed to determine the degree of filling of the pores up to the edge (via the surface roughness, the greater the roughness, the lower the degree of filling up to the edge of the scaffolds and vice versa) (see Fig. 7).

### Drug release experiments

#### CLI release

**Fig. 8:**
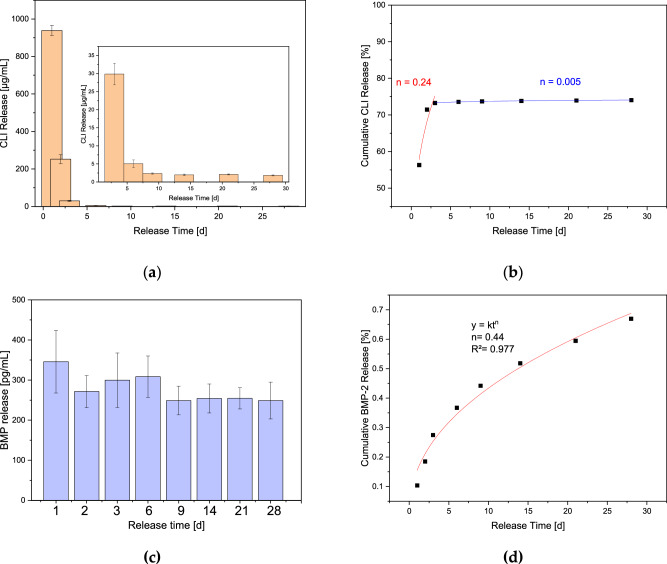
Dual release of **a**, **b** clindamycin and **c**, **d** BMP-2; the small image in (**a**) represents the enlargement of the released concentration from days 3 to 28; **b**, **d** cumulative release for CLI or BMP-2 fitted according to Ritger [51], the diffusion coefficient *n* = 0.43 represents Fickian diffusion; 0.43 < *n* < 1 and *n* < 0.43 represents anomalous transport: *n* = 1 zero-order release for cylindric samples

The CLI was released with a burst release of 939 ± 27 µg/mL, which corresponded to a value of 55% on day 1 based on the initial concentration of the gel and the loading volume of 100 µL. The further transport was anomalous (non-Fickian) with a diffusion coefficient of *n* = 0.24 up to day 3 and thereafter with *n* = 0.005 (corresponding to Ritger [51]) see Fig. 8. Further release occurred until day 28 with a value of 1.83 ± 0.13 µg/mL, which was still well above the MIC for CLI against *Staph. aureus* with 0.06 µg/mL.

#### BMP-2 release

Apart from the burst release on day 1, the BMP-2 release is almost constant over the 28 days with values between 250 and 300 pg/mL (Fig. 8b). The cumulative release corresponds approximately to Fickian diffusion with a diffusion coefficient of *n* = 0.44 for cylindrical samples (Fig. 8d).

### Biocompatibility

#### Live dead assay

**Fig. 9:**
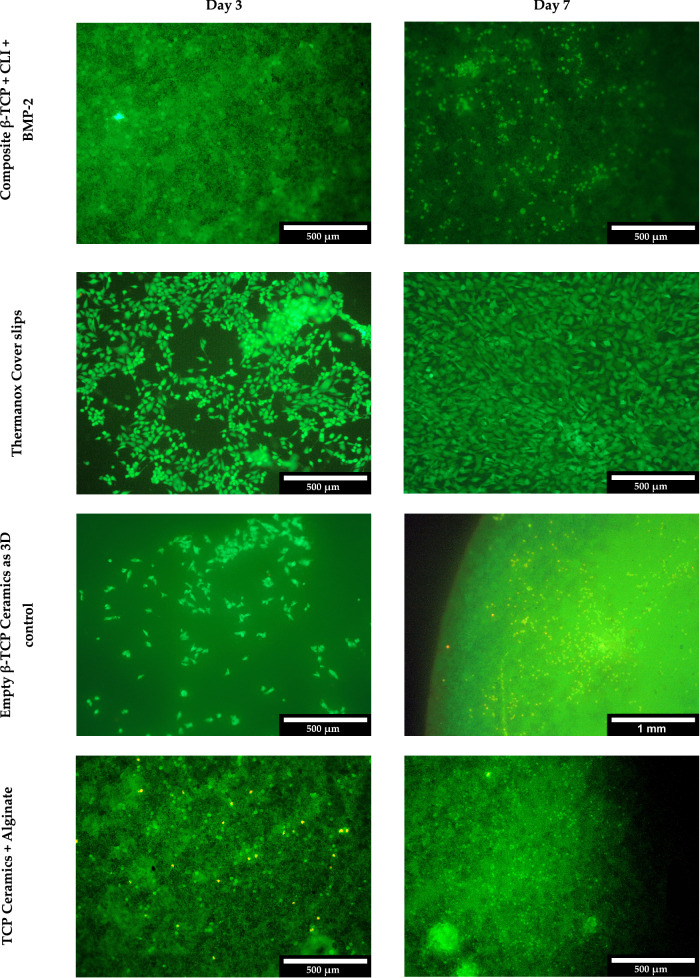
Exemplary comparison of live/dead assay on day 3 and day 7 for composite and control

In the live/dead assay, an increase in MG-63 cells was observed over time. As expected, however, the increase was lower than with the Thermanox Coverslip as a control. Figure 9 shows examples of the composite and control after 3 and 7 days.

#### Cell proliferation

**Fig. 10:**
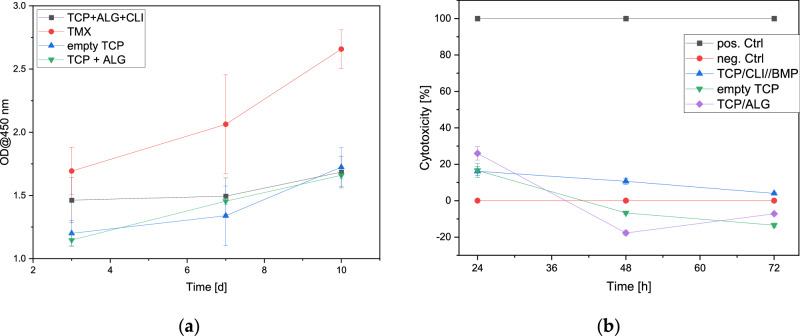
Cell proliferation (**a**) and cytotoxicity (**b**) of the composites compared to the 2D control on the Thermanox Coverslip, empty β-TCP and alginate-filled β-TCP

In the cell proliferation study, the metabolic activity of the MG-63 cells was measured over time. A measurement could be made for all time points. The metabolic activity increases for both the composites and the control (Thermanox Coverslips). Figure 10a shows the cell proliferation after 3, 7 and 10 days in comparison to the control.

#### Cytotoxicity

The cytotoxicity of the composite revealed no negative effects on the MG-63 cells. A slight foreign body effect can be observed on day 1 with values in the region of 20%. However, this is relativized again on days 2 and 3, as can be seen in Fig. 10b. Here the values drop to 0% (negative values also correspond to 0% cytotoxicity).

### Testing MIC

The MIC was determined for each day of the release experiment, whereby the released concentrations were analyzed natively and in dilutions of 4, 2, 1, 0.5; 0.25; 0.12; 0.06; 0.03; 0.015 and 0.008 µg/l. The dilutions were made based on the HPLC readings. For releases smaller than the dilution range, dilutions were always made to the next smallest value. This results in an MIC between 0.03 and 0.12 µg/ml for the first 6 days. From day 9, the MIC increases from 0.5 µg/ml to the undiluted measured value. This means that all concentrations released over the 28 days were antimicrobial. An overview of the MIC values determined is summarized in Table 3.

**Table 3:** Minimum inhibitory concentration of the released clindamycin

Sample	Clindamycin concentrations [µg/mL]												
Concentration	4	2	1	0.5	0.25	0.12	0.06	0.03	0.015	0.008	GC	EV	MIC
D1	−	−	−	−	−	−	−	−	+	++	+++	−	0.03
Concentration	4	2	1	0.5	0.25	0.12	0.06	0.03	0.015	0.008	GC	EV	MIC
D2	−	−	−	−	−	−	−	+	++	+++	+++	−	0.06
Concentration	4	2	1	0.5	0.25	0.12	0.06	0.03	0.015	0.008	GC	EV	MIC
D3	−	−	−	−	−	−	+	++	+++	+++	+++	−	0.12
Concentration	2	1	0.5	0.25	0.12	0.06	0.03	0.015	0.008	0.004	GC	EV	MIC
D6	−	−	−	−	−	+	++	+++	+++	+++	+++	−	0.12
Concentration	2	1	0.5	0.25	0.12	0.06	0.03	0.015	0.008	0.004	GC	EV	MIC
D9	−	−	−	+	++	+++	+++	+++	+++	+++	+++	−	0.5
Concentration	2	1	0.5	0.25	0.12	0.06	0.03	0.015	0.008	0.004	GC	EV	MIC
D14	−	−	+	++	+++	+++	+++	+++	+++	+++	+++	−	1
Concentration	2	1	0.5	0.25	0.12	0.06	0.03	0.015	0.008	0.004	GC	EV	MIC
D21	−	+	+	+	++	++	+++	+++	+++	+++	+++	−	2
Concentration	2	1	0.5	0.25	0.12	0.06	0.03	0.015	0.008	0.004	GC	EV	MIC
D28	−	+	+	+	++	++	+++	+++	+++	+++	+++	−	2

*GC* growth control, *EV* empty value

## Discussion

The characterization of the ceramics by EDX showed a Ca/P ratio of 1.5 and the XRD measurements revealed 99.6% phase-pure β-TCP with traces of CPP. Furthermore, a pore size of 4.2 ± 0.6 µm and 46.5 ± 3.6% total porosity could be determined. The composition and porosity of the ceramic thus correspond to our specifications and are comparable with the preliminary work of Mayr et al. [54] and Seidenstuecker et al. [48].

The GPC analyses of the alginate revealed a reduction in the molar mass, i.e., the chain length of the alginate after plasma sterilization. Cardoso et al. [55] also reported a reduction in the molar mass of alginate, to varying degrees for different sterilization processes. With regard to the planned use for loading the porous ceramics and as an active ingredient carrier, it was found that the batch of A2033 had a slightly lower molar mass for the native alginate and a higher molar mass for the plasma sterilized alginate than in the previous studies by Seidenstuecker et al. [48] and Kissling et al. [56]. Since alginate is a natural product, fluctuations in the molar mass and composition are to be expected. Ultimately, the ceramics could be loaded with alginate within 10 min. In comparison to Seidenstuecker et al. [48], there was only a difference in the concentration of 2% w/v instead of 2.5% w/v – but the viscosity was significantly higher at 1647 mPas compared to 550 mPas. Similar to Seidenstuecker et al., Ritschl et al. and Kissling et al. [48, 49, 56], the ceramic could be fully loaded, which could be visualized very well using fluorescence microscopy. The interaction of total porosity, pore diameter and alginate filling plays an important role in drug release. As we have already shown, the type of porosity (micro vs. macro) also plays an important role in this context, especially if the scaffolds are to be load-bearing. The compressive strength is directly proportional to the total porosity and the pore diameter [57].

Despite the higher initial viscosity of the alginate, the release kinetics are different from those of Seidenstuecker et al. [48] with the same pore diameter and total porosity. However, this is due to the fact that vancomycin was released in that preliminary work and CLI in the present case. Vancomycin has a molar mass of 1449.3 g/mol and CLI 424.98 g/mL, this also explains the higher burst release of 55% compared to 35% for Seidenstuecker et al. [48]. Despite all the differences, a release over 28 days with measured values above the respective MIC against *Staph.* *aureus* was achieved, as with Seidenstuecker. As with Seidenstuecker, the release behavior was an anomalous transport in relation to the nomenclature of Ritger et al. [51]. Nevertheless, all CLI concentrations released over 28 days were antimicrobial. The release of BMP-2, on the other hand, showed almost constant values, which can be explained by Fick’s diffusion if the diffusion coefficient is considered. The BMP-2 release differs greatly from that described by Kissling et al. [56]. This may again be due to the alginate. Unfortunately, no investigation of Kissling’s diffusion mechanism was carried out, so that no comparison is possible in this respect. However, Zurlinden et al. [58] were able to demonstrate comparable releases with the covalent binding of rhBMP-2 to calcium phosphate granulate.

The biocompatibility tests were carried out in three stages. First, live/dead staining was performed after 3, 7 and 10 days. This showed a steady increase in the number of cells and very few dead cells in the microscopic images. Compared to the 2D control, the growth was not quite as pronounced, but this is not surprising. Fontoura et al. [59] also report differences in cell number and cell morphology when comparing 2D and 3D cell cultures. The proliferation measurements using WST-I showed similar results to the live/dead staining – an increase in proliferation over time with differences to the 2D growth control on the Thermanox Coverslip. Cytotoxicity measurements showed decreasing values of cytotoxicity from days 1 to 3. The highest value was not the composite with CLI and BMP-2 but the ceramic with alginate without active ingredients. Seidenstuecker et al. and Ritschl et al. [48, 49] also observed similar cytotoxicity progressions.

The determination of the MIC showed that all released concentrations were antimicrobially effective. In most cases, the dilutions also yielded the MIC against *Staph.* *aureus* according to EUCAST [52] with values between 0.03 and 0.12 µg/mL. The concentrations released from day 9 showed higher MICs, similar to a previous study [48].

## Limitations of the study

Despite taking precautions to minimize exposure to light during the preparation of gels containing CLI and the subsequent measurements, there remains a possibility of light-induced damage, particularly in the microtiter experiments. This concern is heightened, especially at the lower concentrations observed toward the conclusion of the experiments, even though efforts were made to handle the light-sensitive CLI with care.

## Conclusions

In summary, we can say that we have achieved our goal. The β-TCP ceramics produced were in line with our preliminary work with around 5 µm pore diameter and 40% total porosity. We were able to release CLI for 28 days and the concentrations were all antimicrobial. BMP-2 was released at the same time. The release kinetics for CLI corresponded to anomalous transport, whereas for BMP-2 Fick’s diffusion was present. The released concentrations of CLI and BMP-2 had no negative effects on the MG-63 cells used in the biocompatibility studies. This means that our approach can be used in the future as a replacement for PMMA chains that still contain gentamicin. Our composite is fully biodegradable and does not require a second surgery for removal or replacement. In addition, the antibiotics can be adapted to the patient and his or her microbiogram.

## Data Availability

The data presented in this study are available on request from the corresponding author.
